# Prevalence of Hemoglobin-S and Baseline Level of Knowledge on Sickle Cell Disease Among Pregnant Women Attending Antenatal Clinics in Dar-Es-Salaam, Tanzania

**DOI:** 10.3389/fgene.2022.805709

**Published:** 2022-04-11

**Authors:** Hilda J. Tutuba, Agnes Jonathan, William Lloyd, Fredrick Luoga, Emanuela Marco, Joyce Ndunguru, Benson R. Kidenya, Julie Makani, Paschal Ruggajo, Irene K. Minja, Emmanuel Balandya

**Affiliations:** ^1^ Sickle Pan-African Research Consortium (SPARCO), Dar-es-Salaam, Tanzania; ^2^ Sickle Cell Program, Department of Hematology and Blood Transfusion, Muhimbili University of Health and Allied Sciences (MUHAS), Dar-es-Salaam, Tanzania; ^3^ Department of Physiology, MUHAS, Dar-es-Salaam, Tanzania; ^4^ Department of Biochemistry and Molecular Biology, Catholic University of Health and Allied Sciences- Bugando, Mwanza, Tanzania; ^5^ Department of Internal Medicine, MUHAS, Dar-es-Salaam, Tanzania; ^6^ Department of Restorative Dentistry, MUHAS, Dar-es-Salaam, Tanzania

**Keywords:** sickle cell disease, knowledge, pregnant women, maternal screening for SCD, hemoglobin-S, health education, antenatal clinic, awareness of SCD

## Abstract

**Background:** Sickle cell disease (SCD) is the single most important genetic cause of childhood mortality globally. Newborn screening (NBS) is the recommended intervention aimed at early identification of babies with SCD and their linkage to care. To ensure success of NBS, pregnant women need to have the required knowledge on SCD and therefore motivation to screen their babies.

**Objective:** The aim of this study was to determine the prevalence of hemoglobin-S and assess the baseline level of knowledge on SCD among pregnant women attending antenatal clinics in urban settings in Dar-es-Salaam, Tanzania.

**Methods:** This cross-sectional study was conducted between August 2020 and February 2021, involving 600 pregnant women at 20–28 weeks of gestation attending antenatal clinics at Buguruni Health Center, Mbagala Hospital, and Sinza Hospital in Dar-es-Salaam, Tanzania. We administered a structured questionnaire to all participants to assess socio-demographic characteristics and baseline level of knowledge on SCD, where those scoring 7 or higher out of 10 questions were considered to have good knowledge. We screened for SCD a total of 300 participants from two centers (Buguruni Health Center and Mbagala Hospital) by using Sickle SCAN point-of-care test (BioMedomics Inc., United States). We used SPSS version 23 to analyze the data. On determining the association between level of knowledge and socio-demographic factors, we used Pearson’s Chi-square and multivariate logistic regression in ascertaining the strength of associations.

**Results:** Of the 600 participants, the majority were of the age between 26 and 35 years (51%), with the parity of 1-3 children (55.8%) and secondary level of education (43%), while 56% were self-employed. Only 14.7% had good knowledge on SCD. The majority of the participants had ever heard of SCD (81.3%), most of them heard from the streets (42.4%), and only 2.4% heard from hospitals. Of all 600 study participants, only 2 (0.3%) knew their SCD status while 7.7% declared having a family history of SCD. A proficient level of knowledge on SCD is associated with a high level of education, occupation, and knowing personal status of SCD. Among 300 participants who were screened for SCD, 252 were Hb-AA (84%), 47 were Hb-AS (15.7%), and 1 (0.3%) was Hb-SS.

**Conclusion:** Despite the high prevalence of hemoglobin-S among pregnant women attending antenatal clinics in urban settings in Tanzania, there is a poor level of knowledge on SCD and personal knowledge of SCD status. Maternal screening and health education on SCD should be included as part of the comprehensive package for health promotion at antenatal clinics.

## Introduction

Sickle cell disease (SCD) is the single most important genetic cause of childhood mortality globally. Worldwide, it is estimated that about 400,000 babies are born with SCD each year, with the greatest burden being from sub-Saharan Africa where more than 75% of all sickle cell disease occurs, with this proportion projected to increase by 2050 (Piel et al., 2013). The sickle cell trait (SCT) and disease are more prevalent in sub-Saharan Africa; in Benin, the prevalence is estimated to be 25%, in Nigeria it ranges from 24 to 25%, and in Uganda the trait manifests in up to 30% of the population (Rahimy et al., 2003; Ndeezi et al., 2016). Tanzania has one of the highest annual births of SCD individuals in the world, estimated to reach 11,000 births a year, while the prevalence of sickle heterozygous state in Tanzania is between 13 and 20% (Ambrose et al., 2018; Nkya et al., 2019). In the absence of care, the majority of children with SCD succumb early on in life due to severe anemia, vaso-occlusive crisis, and invasive bacterial infection such as pneumonia, septicemia, and meningitis (Nwabuko et al., 2016; Manuscript, 2017).

The World Health Organization (WHO) recommended a set of public health interventions to reduce the burden of SCD which includes increased awareness, early diagnosis, and improving the quality of health care to affected individuals. Measures to prevent disease severity should be taken through the counseling of individuals with high risk of having a child with SCD (Clavagnier, 2012; Manuscript, 2017).

Health promotion refers to any activity that aims to achieve better health in a community. It includes health education, disease prevention, and health screening (Al-Ateeq and Al-Rusaiess, 2015). Health education is an important component of health promotion where medical and other health professionals provide information, advice, counseling, reassurance, and support in order to help people adopt behaviors that will enable them to control and change their lifestyles in order to improve their health (Al-Ateeq and Al-Rusaiess, 2015).

Antenatal care (ANC) is a major platform for health promotion among pregnant women. In Tanzania, the care, that is, provided during antenatal visits, include identification and management of obstetric complications such as preeclampsia, tetanus toxoid immunization, intermittent preventive treatment for malaria during pregnancy (IPTp), as well as identification and management of infections such as HIV, syphilis, and other sexually transmitted infections (Clavagnier, 2012). Studies have shown that early identification of patients and linkage to care can significantly reduce the morbidity and mortality associated with SCD. Particularly, newborn screening for SCD has had a major impact in overturning the morbidity and mortality associated with SCD in Europe and the United States (Nwabuko et al., 2016; Marcheco-Teruel, 2019).

With regards to health education, information given during ANC visits in Tanzania mostly aims to promote attendance to health facilities at birth and health behaviors such as breastfeeding, early postnatal care, and planning for optimal pregnancy spacing (Kearns et al., 2014; Al-Ateeq and Al-Rusaiess, 2015). Currently in our settings, no SCD health education or screening is provided at ANCs, also no similar study has been conducted to assess the prevalence of hemoglobin S and baseline level of knowledge among pregnant women attending antenatal clinics in Dar-es-Salaam.

ANCs are an important platform for health promotion in Tanzania. With approximately 11,000 babies born with SCD each year, it is important to evaluate the feasibility of integrating SCD screening services in ANCs in Tanzania. Education and screening for SCD to pregnant women during ANC visits will help women know if they are among the 13–20% of the population with sickle cell trait (SCT) (Ambrose et al., 2018; Nkya et al., 2019), also be aware of the importance of screening their newborn babies as they may either have SCD or SCT despite them not having the disease. Furthermore, it will empower mothers of children who will be born with SCD to know the importance of early attendance to SCD clinics. Ultimately, this will increase in the number of newborns with SCD who are identified and linked to care early on in life, leading to the reduction in the morbidity and mortality due to SCD in Tanzania.

Our study team at the Sickle Cell Programme, Department of Haematology and Blood Transfusion, Muhimbili University of Health and Allied Sciences (MUHAS) in Dar-es-Salaam has been at the forefront of conducting research on SCD in Tanzania. Over the years, research from our team has delineated various aspects including clinical epidemiology, health systems, research ethics, and genomic profiles of SCD in Tanzania (Makani et al., 2011; Costa et al., 2021; Jacob et al., 2020; Bukini et al., 2020; Masamu et al., 2020; Nkya et al., 2020; Urio et al., 2020). Since 2017, the Sickle Cell Programme is part of the SickleInAfrica consortium, funded by the United States National Institutes of Health (NIH) through the National Heart, Lung and Blood Institute (NHLBI), with the aims to foster connectedness among stakeholders, develop an electronic database of patients, advance standards of care, provide training, and conduct research on pertinent areas on SCD in the sub-Saharan African context (Makani et al., 2020).

## Materials and Methods

Study design and setting: This cross-sectional study involved 600 pregnant women at Buguruni Health Center, Mbagala Hospital, and Sinza Hospital. All are public health facilities in Dar-es-Salaam, Tanzania. The antenatal clinics from these health facilities are conducted from Monday to Friday every week with exception of public holidays and provide different services including vaccination, Prevention of Mother to Child Transmission (PMTCT), family planning, and health education (mostly on danger signs in pregnancy, hypertension in pregnancy, anemia in pregnancy, and Rhesus incompatibility, for about 1 h per session) but no education is given on SCD. Mondays and Wednesdays are the clinic days for those attending for the first time, while Tuesdays, Thursdays, and Fridays are for those making a follow-up visit. About 50 to 100 pregnant women attend an ANC every day at each health facility.

There are neither SCD clinics nor newborn screening services in any of the study sites though the facilities provide post-natal immunization services. All the study sites are within 1–10 km away from their respective regional referral hospitals which are Amana, Temeke, and Mwananyamala where SCD clinics are conducted weekly.

Study participants: This study involved pregnant women who were attending antenatal clinics at the respective sites. This study population was selected because there was no study done to assess the prevalence of hemoglobin S and the baseline level of knowledge among this group.

### Sample Size and Sampling Technique

The formula for sample size calculation was

m1=[Zn2(r+1)P(1−P)+Zβ+rp0(1−P0)+p1(1−p1)]2r(p0−p1)2

where: p=P0+rp1r+1

P^0^ is the proportion in the population that received health education only, and P^1^ is the proportion in the population that received health education and maternal screening, r is the case and control ratio.

Assumptions: alpha = 0.05 (two sided), Power = 95%, P^0^ = 0.5, P^1^ = 0.35, **m0/m1** = r = 1

Attrition rate = 20%

M1 = 220.

The minimum sample size for this study was 440 participants, of which a minimum of 220 were to be screened and receive health education while a minimum of 220 were to receive only health education. Participants were selected by using a convenient sampling technique where pregnant women of gestation age between 20 and 28 weeks, with no complications that risk termination of pregnancy (such as heart failure, pregnancy-induced hypertension, threatened abortion, hyperemesis gravidarum, and gestational diabetes) were enrolled. Also, participants included pregnant women who had no history of blood transfusion within the past 4 months prior to enrollment as the donor blood may interfere with the results of the SCD screening test (this was applicable to those of the screening population). A total of 160 more pregnant women were added beyond the minimum sample size for a final total of 600 participants.

### Collection of Socio-Demographic Data and Knowledge on Sickle Cell Disease

We conducted this study from August 2020 to February 2021, whereby 600 pregnant women who attended antenatal clinics at the respective sites and met the criteria were enrolled in the study. Following written informed consent, we administered a structured questionnaire to all 600 participants collecting the socio-demographic information and assessed the knowledge on SCD through a series of 10 questions. The questions assessed, the attitude on relating SCD to blood cancer and life expectance of SCD patients, knowledge on the mode of acquiring and diagnosis of SCD as well as the most common signs and symptoms of SCD. After administration of the questionnaire, we provided the health education on SCD to all 600 participants.

### Maternal Screening for Sickle Cell Disease

**FIGURE 1:**
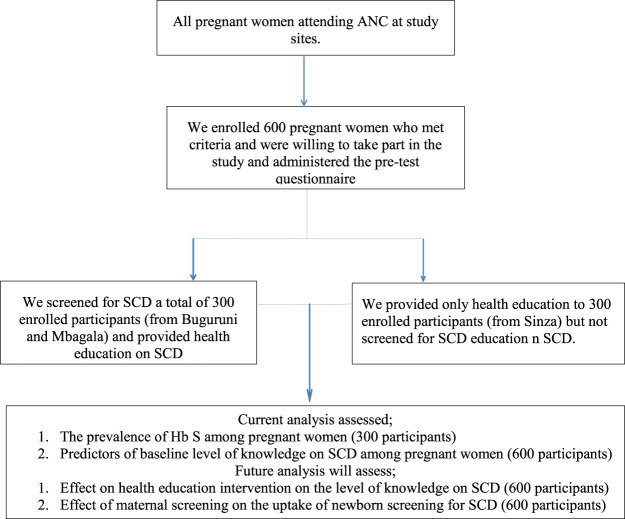
Summary of data collection procedure.

Following collection of socio-demographic information, we counseled and then screened for SCD a total of 300 participants from two sites (Buguruni Health Center and Mbagala Hospital) by using Sickle SCAN® point-of-care test, which is a rapid, qualitative, lateral flow immunoassay kit for the identification of sickle cell disorder of hemoglobin A, S, and C (BioMedomics Inc., United States). We conducted the screening at antenatal laboratories where other screening activities took place (HIV, HB level). A small amount of whole blood (5 μl) was taken by finger prick using the provided capillary sampler. The sampler was placed into the buffered loaded pretreatment module to release hemoglobin by lysing erythrocytes. Three drops of the treated sample were dropped from the pretreatment module and added to the sample inlet of the Sickle SCAN® cartridge. Results were read within 5 min. The presence of hemoglobin variants A and S was indicated by blue lines in their designated regions. The remaining 300 patients from Sinza Hospital were not screened for SCD (Figure 1). This will allow future assessment of the uptake of newborn screening for SCD among babies born to mothers who were screened (300 participants) compared to those who were not screened (300 participants).

### Statistical Analysis

We used Statistical Package for the Social Sciences (SPSS) version 23 for data analysis. We obtained the prevalence of S-hemoglobin as the number of screened participants with Hb AS plus those with Hb SS divided by the number of all who were screened (Hb AA + Hb AS + Hb SS) times 100 ((Hb AS + Hb SS)/(HbAA + Hb AS + Hb SS) * 100).

We expressed the descriptive statistics such as participant characteristics in frequencies and presented in tables. We graded the level of knowledge depending on the number of correct responses scored from the questionnaires which was constructed from different literature which assessed the level of knowledge on SCD in different groups including pregnant women, also modified to fit the local context (Babalola et al., 2019; Obed et al., 2017a; Al-Qattan et al., 2019). A correct answer was scored as “1” while a wrong response was scored as “0”. Total knowledge score ranging from 0 to 10 was calculated as the sum of all correct answers considering the following score range; < 7 poor knowledge, 7–10 good knowledge.

We did inferential statistics on factors associated with level of knowledge by comparing the different categories between the dependent variable (overall knowledge on SCD) with various independent variables (age, parity, marital status, level of education, occupation, if ever heard of the term SCD, the source of information on SCD, personal status, and family history on SCD) using Chi square test (for independent variables with all expected values ≥ 5). We considered a two-tailed p-value below 0.05 to be statistically significant. Subsequently, we did univariate and multivariate binomial logistic regression to test the strength of association between the main dependent variable (overall level of knowledge) with the independent variables. We entered into multivariate logistic regression only independent variables with p-values ≤ 0.2 in univariate analysis (level of education, occupation, and family history of SCD) to adjust for the effect of multiple predictors. We used the odds ratio to explain the relationship between dependent variables and associated factors and used a confidence level at 95% in determining statistical significance.

Besides delineation of the baseline level of knowledge and predictors of knowledge on SCD (all 600 participants), current analysis also reported on the prevalence of hemoglobin S among pregnant women who were screened (300 participants). In the future, we will assess the effect of health education intervention on the level of knowledge on SCD as well as the uptake of newborn screening for SCD among babies born to mothers who were screened (300 participants) compared to those who were not screened (300 participants).

### Ethical Consideration

We obtained ethical clearance for this study from Muhimbili University of Health and Allied Sciences research ethics committee with certificate number **MUHAS-REC-08-2020-333**. We obtained the permission to conduct the study from Regional Medical Officer- Dar-es-Salam, the District medical officers from Ilala, Ubungo, and Temeke districts, as well as the respective hospitals, Buguruni Health Center, Sinza Hospital, and Mbagala Hospital. We obtained the written informed consent from every participant. Following collection of socio-demographic information and baseline knowledge on SCD, all participants received health education on SCD, the importance of screening their babies for SCD, and were also informed on the available SCD services in Dar-es-Salaam. Participants who were screened for SCD were informed of their SCD status. One participant who was found to be HbSS was encouraged to start clinic at her respective regional referral hospital.

## Results

### Demographic Characteristics of Participants

We enrolled a total number of 600 pregnant women from the antenatal clinics in the study. The majority of participants were in the age range of 26–35 (51.0%), with parity of 1–3 children (55.8%). Furthermore, most of the participants were married 495 (82.5%), self-employed 340 (56%), and had a secondary level of education 250 (43%) (Table 1). A total of 488 participants (81.3%) reported to have heard of the term SCD. The major sources of information on SCD were the streets 207 (42%) and schools 121 (24.8%) and only 12 participants (2.4%) heard of SCD at the hospital. Moreover, 46 participants (7.7%) reported having a family history of SCD and only 2 (0.3%) knew of their SCD status (Table 1).

**TABLE 1:** Socio-demographic characteristics of pregnant women attending antenatal clinics in Dar-es-Salaam (N = 600).

Pregnant women characteristics	n	Percent (%)
Age (years)		
<18	2	0.3
18–25	217	36.2
26–35	306	51.0
>35	75	12.5
Parity		
0	243	40.5
1–3	335	55.8
>3	22	3.7
Marital status		
Married	495	82.5
Not married	105	17.5
Level of education		
Illiterate	19	3.2
Primary level	249	41.5
Secondary level	258	43.0
College/university	74	12.3
Occupation		
Employed	65	10.8
Self-employed	340	56.7
Homemaker	195	32.5
Heard of the term SCD		
Yes	488	81.3
No	112	18.7
Source of information on SCD (N = 488)		
School	121	24.8
Media	72	14.8
Home	76	15.6
Street/peers	207	42.4
Hospital	12	2.4
Knowing personal status		
Yes	2	0.3
No	598	99.7
Family history of SCD		
Yes	46	7.7
No	554	92.3

### Prevalence of S-Hemoglobin Among Pregnant Women

Table 2 shows results of maternal SCD screening among 300 pregnant women from Sinza Health Center and Mbagala Hospital. Most of the participants were Hb-AA 252 (84%). There were 47 participants (15.7%) who had sickle cell trait and one participant (0.3%) had Hb-SS. None of the participants had Hb-SC.

**TABLE 2:** The prevalence of hemoglobin-S among pregnant women attending antenatal clinics in Dar-es-Salaam (N = 300).

Sickle cell status	Frequency (n)	Percent (%)
HbAA	252	84.0
HbAS	47	15.7
HbSS	1	0.3
TOTAL	300	100

### Overall Level of Knowledge on Sickle Cell Disease Among the Study Participants

Only 88 participants (14.7%) had good overall knowledge on SCD. From the individual questions which assessed the attitude, mode of acquiring, and diagnosis of SCD, only 22% believed that SCD is different from blood cancer while only 28% believed that individuals with SCD can live to normal life expectancy. Almost half of the participants (48%) knew that SCD is an inherited disease, only 27.7% knew that phenotypically normal parents may have a child with SCD in case both parents have sickle cell trait. On assessing the level of knowledge on the common signs and symptoms of SCD, anemia was the most known sign (49.8%), while pain was known to only 25.7% of the participants, with the least one being dactylitis (13%). Other symptoms assessed were jaundice known to only 26.7% and abdominal distension due to enlarged spleen known to only 17.5% of the participants (Table 3).

**TABLE 3:** Response to questions on knowledge on SCD.

Question	True answer	Correct responses n (%)	Wrong responses n (%)
SCD is the same as blood cancer.	No	132 (22)	468 (78)
Do the lives of SCD patients have an age limit?	No	172 (28.7)	428 (71.3)
Mode of acquiring SCD.	Inheritance	290 (48.3)	310 (51.)
What is the mode of diagnosing SCD?	Blood test	179 (29.8)	421 (70.2)
Can the phenotypically normal parents get a child with SCD?	Yes	166 (27.7)	434 (72.3)
Are the following signs and symptoms of SCD?			
Anemia	Yes	299 (49.8)	301 (50.2)
Pain	Yes	154 (25.7)	446 (74.3)
Jaundice	Yes	160 (26.7)	440 (73.3)
Abdominal distention due to splenomegaly	Yes	105 (17.5)	495 (82.5)
Dactylitis	Yes	82 (13.7)	518 (86.3)

### Factors Influencing Knowledge on Sickle Cell Disease Among Pregnant Women Attending Antenatal Clinics in Dar-Es-Salaam

In ascertaining the association between the overall level of knowledge on SCD with participants’ characteristics, we observed that the overall level of knowledge was associated with participant’s level of education (*p*-value < 0.001), occupation (*p*-value 0.014), and knowing their personal status of SCD (*p*-value 0.021). Among those who had ever heard of SCD, the level of knowledge was associated with the source of information on SCD (*p*-value < 0.001), which was higher among those who heard of SCD from school and home compared to those who heard from the streets. Other participant characteristics such as age, parity, marital status, and having a family history of SCD were not found to be associated with having good knowledge on SCD (Table 4).

**TABLE 4:** Socio-demographic characteristics and overall level of knowledge on SCD among pregnant women attending antenatal clinics in Dar-es-salaam (N = 600).

Characteristic	n (%)	Overall knowledge on SCD N = 600		*p*-value (chi-square test)
		Poor (<7) N = 512 (85.3%) n (%)	Good (≥7) N = 88 (14.7%) n (%)	
Age (years)	—	—	—	0.687^a^
<18	2 (0.3)	2 (100.0)	0 (0)	—
18–25	217 (36.2)	188 (86.6)	29 (13.4)	—
26–35	306 (51.0)	257 (84.0)	49 (16.0)	—
>35	75 (12.5)	65 (86.7)	10 (13.3)	—
Parity	—	—	—	0.378^a^
0	243 (40.5)	202 (83.1)	41 (16.9)	—
>3	22 (3.7)	20 (90.9)	2 (9.7)	—
1–3	335 (55.8)	290 (86.6)	45 (13.4)	—
Marital status	—	—	—	0.430
Married	495 (82.5)	425 (85.9)	70 (14.1)	—
Not married	105 (17.5)	87 (82.9)	18 (17.1)	—
Level of education	—	—	—	**<**0.001
Illiterate/primary	268 (44.7)	249 (92.9)	19 (7.1)	—
Secondary	258 (43.0)	207 (80.2)	51 (19.8)	—
College/university	74 (12.3)	56 (75.7)	18 (24.3)	—
Occupation	—	—	—	0.014
Employed	65 (10.8)	52 (80.0)	13 (20.0)	—
Self-employed	340 (56.7)	282 (82.9)	58 (17.1)	—
Homemaker	195 (32.5)	178 (91.3)	17 (8.7)	—
Source of information (N = 488)	—	—	—	**<**0.001**^a^**
School	121 (24.8)	89 (73.6)	32 (26.4)	—
Media	72 (14.8)	61 (84.7)	11 (15.3)	—
Home	76 (15.6)	58 (76.3)	18 (23.7)	—
Street	207 (42.4)	182 (87.9)	25 (12.1)	—
Hospital	12 (2.4)	10 (83.3)	2 (16.7)	—
Knowing personal status	—	—	—	0.021^b^
Yes	2 (0.3)	0 (0.0)	2 (100)	—
No	598 (99.7)	512 (85.6)	86 (14.4)	—
Family history of SCD	—	—	—	0.159
Yes	46 (7.7)	36 (78.3)	10 (21.7)	—
No	554 (92.3)	476 (85.9)	78 (14.1)	—

a = Likelihood ratio.

b = Fisher’ exact test.

Table 5 summarizes results of univariate and multivariate logistic regression analysis. The final model revealed a strong association between overall level of knowledge on SCD with level of education of participants and on participant’s occupation. The odds of having good knowledge on SCD among pregnant women with college/university level of education was 3.9 times higher than those who were illiterate/primary level (AOR = 3.94; 95% CI = 1.89–7.94). Also, those having a secondary level of education had 2.9 times higher odds of having good knowledge than the illiterate/primary level of education (AOR = 2.94; 95% CI = 1.67–5.18). Occupation was also seen to be associated with the level of knowledge on SCD among pregnant women whereby those who were self-employed had 1.8 times higher odds of having good knowledge than those who were homemakers (AOR = 1.84; 95%CI = 1.02–3.29).

**TABLE 5:** Regression analysis of factors influencing knowledge of SCD among pregnant women attending ANC in Dar-es-Salaam.

Factors	n (%)	Univariate analysis COR ((95% CI))	Multivariate analysis AOR ((95% CI))
Age			
<18	2 (0.3)	*	—
18–25	217 (36.2)	1.00 (0.46–2.17)	—
26–35	306 (51.0)	1.24 (0.60–2.58)	—
>35	75 (12.5)	References	—
Parity			
0	243 (40.5)	2.03 (0.46–9.02)	—
1–3	335 (55.8)	1.55 (0.35–6.87)	—
>3	22 (3.7)	References	—
Marital status			
Married	495 (82.5)	0.80 (0.45–1.40)	—
Not married	105 (17.5)	References	—
Level of Education			
Illiterate/Primary	268 (44.7)	References	—
Secondary	258 (43.0)	3.23 (1.85–5.64)	2.94 (1.67–5.18)
College/university	74 (12.3)	4.21 (2.08–8.54)	3.88 (1.89–7.94)
Occupation			
Employed	65 (10.8)	2.62 (1.19–5.74)	1.83 (0.82–4.10)
Self-employed	340 (56.7)	2.15 (1.21–3.82)	1.84 (1.02–3.29)
Homemaker	195 (32.5)	References	—
Family history			
Yes	46 (7.7)	1.70 (0.81–3.55)	2.08 (0.93–4.42)
No	554 (92.3)	References	—
Source of information			
School	121 (24.8)	2.62 (1.46–4.68)	—
Media	72 (14.8)	1.31 (0.61–0.82)	—
Home	76 (15.6)	2.26 (1.15–4.43)	—
Hospital	12 (2.4)	1.46 (0.30–7.03)	—
Street/peers	207 (42.4)	References	—

*Predicts outcome perfectly.

COR, crude odd ratio; AOR, adjusted odd ratio, 95%CI, confidence interval at 95%.

Besides these predictors, we also found the source of information on SCD to be associated with the level of knowledge on SCD during univariate analysis where those who heard of SCD at school had 2.6 times higher odds of having good knowledge compared to those who heard from the streets/peers (COR = 2.62; 95% CI = 1.46–4.68), and also those whose source of information was home (have a family member or have ever lived with an SCD patient) had 2.2 times higher odds of having good knowledge compared to those who heard of SCD from the streets/peers (COR = 2.26; 95% CI = 1.15–4.43). However, this factor was not included in multiple regression due to its collinearity with level of education which was strongly associated with level of knowledge on SCD and also since only a subset of 488 participants who had ever heard of SCD provided responses on the source of information.

## Discussion

### Overall Findings

Adequate maternal level of knowledge on SCD could act as a catalyst for planned parenthood and uptake of newborn screening for SCD, especially in settings where the prevalence of SCD is high. Here, we show strikingly low levels of knowledge on SCD in a population of pregnant women where the prevalence of hemoglobin S was high. The level of knowledge on SCD was associated with level of education, occupation, and knowing personal status of SCD. These findings have implications in planning interventions such as newborn screening for SCD, and uncovers antenatal clinics as a potential platform for providing health education and maternal screening for SCD with the ultimate goal of improving the uptake of newborn screening for SCD.

### Knowledge of Personal Sickle Cell Disease Status

We found high prevalence (16%) of hemoglobin S among pregnant women attending antenatal clinics in Dar-es-Salaam, concordant with the high prevalence in the general population in Tanzania which ranges from 13–20% (Makani et al., 2011; Ambrose et al., 2018). Despite the high prevalence of hemoglobin S and although 46 participants (7.7%) admitted having a family history of SCD, only 2 of 600 (0.3%) participants reported to know their SCD status. Among the 300 participants who were screened for SCD, none knew of her SCD status prior to screening in the current study, including one participant who was found to be Hb-SS. This level of knowledge of personal SCD status is very low compared to that reported by Obed et al. in Ghana where 10% of pregnant women self-reported sickle cell trait, and that reported by Treadwell et al. where 16% reported knowledge of their sickle cell trait status (Treadwell et al., 2006; Obed et al., 2017b). Also, in a study of 147 African-American patients aged 18–50 years seen in an emergency department, 31% knew of their own trait status (Burnham-Marusich et al., 2016). Furthermore, in a cohort of recent tertiary graduates during their National Youth Service Corps (NYSC) in Benin City, Edo state, Nigeria, about 94.6% of the respondents reported to know their SCD carrier status and 80.8% were willing to avoid carrier marriage, the commonest indication for carrier status check being school entry followed by a doctor’s request and premarital screening (Adewoyin et al., 2015). The observed difference in the level of knowledge on personal SCD status, particularly between Tanzania and sites in Ghana and Nigeria, is possibly attributed to SCD being much more common in West Africa than East Africa (Piel et al., 2013; Bukini et al., 2020).

### General Level of Knowledge on Sickle Cell Disease

Tanzania has one of the highest annual births of SCD individuals in the world, estimated to reach 11,000 births a year (Makani et al., 2011), but there is a poor level of knowledge among pregnant women attending antenatal clinics in Dar-es-Salaam. In this study we found that only 14.7% of participants had good knowledge on SCD. This was almost similar to studies done in West Africa, such as that done in Nigeria assessing the level of knowledge among tertiary level graduates which showed that only 17.8% of respondents had good knowledge of SCD (Adewoyin et al., 2015). Also, the other one done in Ghana among university students showed only 7.1% had excellent knowledge on SCD (disease). The finding that only 27.7% of the pregnant women in the study population knew that parents who are phenotypically normal may have children with SCD is concerning as it heralds a major lack of public awareness on the mode of inheritance of SCD and hence lack of consideration of sickle cell trait status among partners prior to the decision to get married or have a baby. This was also seen in a study done aiming to uncover the levels of knowledge, attitude, and practice (KAP) associated with SCD and premarital genetic counseling in 351 Saudi adults which showed that 28.8% had good knowledge on SCD while only 14.8% of participants were aware of SCD inheritance pattern (Al-Qattan et al., 2019).

Particularly in the study setting, since newborn screening is not yet universal in Tanzania, awareness on SCD among pregnant women could have significant impact by motivating mothers to seek newborn screening or early infant diagnosis of SCD for their babies after birth.

### Factors Associated With the Level of Knowledge on Sickle Cell Disease

The level of knowledge among pregnant women in this study was significantly predicted by the level of education which increased with the increase in level of education; as those with college/university level education as well as secondary level of education had higher odds of having good knowledge compared to those of primary/illiterate level. The good level of knowledge on SCD was also associated with hearing of SCD at school and having lived with a patient with SCD and therefore obtained the information from home.

These findings are similar to observations in a study in Nigeria which showed that the level of knowledge on SCD increased with higher levels of education and also mothers who were already caregivers of SCD patients had a higher knowledge score and a better understanding on SCD inheritance than those who had no children with SCD (Babalola et al., 2019). This was again the same as other studies conducted in Nigeria which showed that higher level of education and knowing a relative with SCD or sickle cell trait was significantly associated with high knowledge of SCD (disease; Ezenwosu et al., 2021). Another study done by Obed et al. showed that respondents with at least secondary education scored an average one point higher on the knowledge test than those with lower education, and knowing someone with SCD was associated with a higher level of knowledge than in individuals who did not know any affected individual (Obed et al., 2017b).

Contrary to our study, the study done by Al-Qattan et al. showed that age group is associated with the level of knowledge (Al-Qattan et al., 2019). Also, another study done by Burnham-Marusich et al. showed age and employment were associated with accuracy on self-reported status of SCD, while marital status was not significantly associated (Burnham-Marusich et al., 2016). In our study, age, parity, marital status, and having a family history of SCD were not found to be associated with having good knowledge on SCD.

Our study showed that knowing personal SCD status was associated with having good knowledge on SCD. This had a perfect prediction where all 2 participants who knew their status had a proficient level of knowledge on SCD. This was similar to a study that showed better knowledge on SCD among individuals with knowledge of their sickle cell trait status compared to those who did not know (Obed et al., 2017b). Occupation was another factor that was associated with good knowledge on SCD in this study where those who were self-employed had higher odds of having good knowledge than homemakers did.

### Sources of Information on Sickle Cell Disease

In this study, over 80% of participants reported to have ever heard of the term SCD, with the majority of them having heard from the street/peers), followed by school, through living with a patient with SCD at home, mass media such as television and radio, and at hospitals. This finding is not far different from another study where the majority of participants seemed to get information on SCD from schools (84.6%), media (12.6%), and health center/family and friends (2.9%) (disease), similar to that shown by Adewoyin et al. (2015). Also, another one showed only 10.1% of participants received information on premarital genetic counselling from health care workers (Al-Qattan et al., 2019). Of note, although over 80% of the participants in the present study reported to have heard of SCD, only 14.7% had good knowledge on SCD. This implies inadequate or inappropriate education provided through the reported outlets. Particularly, it is concerning that only 2.4% of participants reported to have heard of SCD at hospitals where the right information is expected to be provided as shown by Ezenwosu et al. (2021).

The fact that SCD health education is not part of the information offered to pregnant women during their regular antenatal visits may be the reason for this and represents a large missed opportunity. In Tanzania, by 2008, 94% of pregnant women made at least one visit to the antenatal clinic and 62% of pregnant women made at least four visits (Clavagnier, 2012). Besides antenatal clinics, mass media may also be an effective platform for educating the population on SCD.

## Conclusion

The prevalence of hemoglobin S was high among pregnant women in Dar-es-Salaam, Tanzania, although most of them have never been screened and were not aware of their SCD status. Despite the high prevalence of hemoglobin S, an overwhelming majority of pregnant women did not have good knowledge on SCD, implying a missed opportunity for planned parenthood and a high likelihood of not soliciting newborn screening for their babies. Most pregnant women seemed to have received information on SCD from the streets which appeared to have been incorrect or inadequate, and a large opportunity is missed to provide proper health education on SCD in hospital settings, particularly antenatal clinics, as well as through mass media. Inclusion of maternal screening and health education for SCD is advocated as part of the comprehensive package for health promotion at antenatal clinics.

## Data Availability

Raw data supporting the conclusions of this article will be made available by the authors upon reasonable request.
